# Multiple imputation Procedure for Record Linkage and Causal Inference to Estimate the Effects of home-delivered meals

**DOI:** 10.23889/ijpds.v9i5.2551

**Published:** 2024-09-18

**Authors:** Roee Gutman, Kali Thomas, Mingyang Shan

**Affiliations:** 1 Brown University; 2 Johns Hopkins School of Nursing; 3 Eli Lilly and Company

To assess the impact of home-delivered meals programs offered by Meals on Wheels (MOW) a dataset that includes enrollment into MOW and healthcare utilization is required. Because of confidentiality restrictions this data is dispersed over MOW programs internal systems and Medicare claims. In the absence of unique identifying identifiers, MOW beneficiaries are linked to Medicare beneficiaries using probabilistic linking algorithm. However, this procedure may erroneously link individuals across the two datasets, which may result in biased treatment effect estimates and suboptimal interval estimates. We propose a two-stage multiple imputation framework to estimate causal effects when the covariates and outcome information are stored in Medicare claims and the treatment assignment is MOW program data. In the first stage, we create multiple datasets in which MOW beneficiaries are linked to Medicare beneficiaries using a Bayesian record linkage technique. In the second stage, we match Medicare beneficiaries that were not enrolled in MOW programs to those that did. Using these matches we multiply imputed for each MOW beneficiary their unobserved healthcare utilization if they did not receive MOW. This procedure propagates the errors in the linking process and the matching process, and can be used to estimate effects of interventions in other linked datasets when there are no unique identifiers.

